# Factors associated with modern contraceptive use among sexually active adolescent girls and young women (15–24): analysis of the Uganda demographic health survey 2016

**DOI:** 10.3389/frph.2026.1794166

**Published:** 2026-04-02

**Authors:** Bruce Tukamushaba, Persis Tikabulamu, Solome Najjuma, Juliet Ntuulo Mutanda

**Affiliations:** Department of Epidemiology and Biostatistics, School of Public Health Makerere University, Kampala, Uganda

**Keywords:** adolescent girls, contraceptive use, family planning, sexual reproductive health, young women

## Abstract

**Background:**

Modern contraceptive use among adolescent girls and young women (AGYW) is crucial for reducing unintended pregnancies, maternal mortality, and unsafe abortions. In Uganda, adolescent fertility and unmet need for contraception remain high despite government efforts to improve access to family planning services. Understanding the factors influencing modern contraceptive use among AGYW is essential for developing targeted interventions.

**Objective:**

To determine the prevalence and factors associated with modern contraceptive use among sexually active adolescent girls and young women (15–24 years) in Uganda, using data from the 2016 Uganda Demographic and Health Survey (UDHS).

**Methods:**

This study employed a cross-sectional design using secondary data from the 2016 UDHS. 4,534 sexually active AGYW were included in the analysis. The outcome variable was modern contraceptive use, categorized as “Yes” (using a modern contraceptive method) or “No” (not using any modern contraceptive method). Multivariable modified Poisson regression was used to identify factors associated with modern contraceptive use, adjusting for survey design characteristics.

**Results:**

The prevalence of modern contraceptive use among AGYW was 32.6%. The age specific modern contraceptive rate (mCPR) was 8.8% for adolescent girls (15–19) and 23.8% for young women (20–24). Key predictors of contraceptive use at multivariable analysis include region, education, wealth, parity, media exposure, recent sexual activity, and number of sexual partners.

**Conclusion and recommendations:**

Despite ongoing efforts, modern contraceptive use among AGYW in Uganda remains low. To improve uptake, interventions should target younger adolescents, enhance access to youth-friendly services, and address regional disparities. Increasing digital family planning messaging and integrating reproductive health education into school curricula could further improve contraceptive use among AGYW. Future research should explore the lived experiences of AGYW and the contextual factors influencing their reproductive health decisions.

## Background

Family planning is a critical component of reproductive health, enabling individuals and couples to achieve their desired number of children and determine the spacing of their pregnancies. Access to and utilization of modern contraceptive methods are essential for reducing unintended pregnancies, maternal and child mortality, and unsafe abortions, particularly in low- and middle-income countries (LMICs) where healthcare resources are often limited (1, 2). Despite global efforts to improve access to family planning services, significant disparities in contraceptive use persist, especially among vulnerable populations such as adolescents, young women, and those living in conflict-affected or resource-poor settings (3, 4).

Sub-Saharan Africa has the highest unmet need for contraception, with nearly 25% of women of reproductive age affected, but the young women are the most affected (5). Uganda is among the countries with the highest unmet need for family planning services in the region. According to the 2022 Uganda Demographic and Health Survey (UDHS), 24% of currently married women and 39% of sexually active unmarried women have an unmet need for contraception, while the contraceptive prevalence rate remains low at 30% (6, 7). Concurrently, teenage pregnancy remains a critical issue, with 23.5% of adolescent girls aged 15–19 having begun childbearing (7).

These challenges are compounded by Uganda’s fragile health system and high fertility rates, which continue to exert pressure on limited health and socioeconomic resources, especially in rural areas that constitute 80% of the population (8, 9). The high rates of unintended pregnancies and unsafe abortions, which account for 28% of maternal deaths annually, further highlight the urgent need to address the gaps in family planning services for AGYW (6).

Although Uganda's policies permit sexually active adolescents and young women to access modern contraceptives without parental or guardian consent (10), the uptake remains low. Several factors contribute to this situation, including limited knowledge of contraceptive methods, fear of side effects, stigma associated with premarital sex, sociocultural and religious norms, and a lack of youth-friendly services (11, 12). Previous studies have also identified determinants such as education level, marital status, parity, partner support, mass media exposure, age, wealth index, and area of residence as factors influencing contraceptive use among young women (1, 13–16).

Sexual and reproductive health (SRH) services are a fundamental human right. The Sustainable Development Goal (SDG) target 3.7 emphasizes universal access to SRH services by 2030 (17). However, many interventions in Uganda have failed to address the specific needs of AGYW, especially in resource-limited and culturally conservative contexts (3, 18). A comprehensive understanding of the specific factors influencing modern contraceptive use among AGYW is necessary to inform more responsive and targeted policies and programs.

This study seeks to fill this gap by examining the prevalence and determinants of modern contraceptive use among sexually active adolescent girls and young women aged 15–24 in Uganda. Using nationally representative data from the 2016 Uganda Demographic and Health Survey, the findings aim to inform evidence-based strategies to improve contraceptive uptake and reproductive health outcomes among this key demographic, contributing to the broader goal of universal access to SRH services.

## Methods

### Study design

This was a cross sectional study using secondary data from the 2016 Uganda Demographic Health Survey.

### Dependent (outcome) variable

The primary variable “modern contraceptive use” was derived from the existing DHS variable on current contraceptive use by method type “v364”, with responses dichotomized into No “not using a method, uses a traditional method, uses a folkloric method” and Yes “uses a modern method.” See Table 1 for description and recoding.

**Table 1:** Description of the outcome variable and the predictor variables.

Study variable	DHS code	DHS label	Variable type	Recoded variable type
wgt	v005	Women's individual sample weights.	Continuous	Continuous
		To use the sample weight divide it by 1000000 before applying the weighting factor.		
v021	v021	Primary sampling unit	Continuous	Continuous
v023	v023	Stratification used in sample design	Continuous	Continuous
AGE	v012	Respondents current age	Continuous	Categorical (1 “15-19”, 2 “20-24”)
Region	v024	Region	Categorical	Categorical (recoded to region cat: 1“central” 2“eastern” 3“northern” 4“western”)
Place of residence	v025	Type of place of residence	Categorical (1 “urban”, 2 “rural”)	Categorical
Religion	v130	Religion	Categorical (Anglican, Catholic, Muslim, seventh day Adventist, orthodox, Pentecostal/born again/evangelical, Baptist, Jehovah's Witness, traditional, other)	Categorical (religion was recoded to 1“anglican” 2“catholic” 3“muslim” 4“pentecostal” 5“others”)
Highest level of education	v106	Highest level of education	Categorical (0 “No education,” 1“ Primary,”2“ Secondary,”3“ Higher”)	Categorical
Wealth index combined	v190	Wealth index combined	Categorical (1 “lowest,” 2 “second,” 3 “middle,” 4 “fourth,” 5 “highest”)	Categorical
Marital status	v501	Current marital status of the respondent	Categorical (0 “Never in union”, 1 “Married”, 2 “Living with partner”, 3 “Widowed”, 4 “Divorced”, 5 “No longer living together/separated”)	Recoded to v501_cat (0 “Never in union”, 1 “Married”, 2 “ Living with a partner”, and 3 “No longer living with a partner”)
Currently working	v714	Respondent currently working	Categorical (0 “No”, 1 “Yes”	Categorical
Parity	v201	Total number of children ever born	Continuous (0–8)	Categorical (recoded to Parity: 1 “No children”, 2 “1–2 children”, 3 “3 or more children”)
Heard family planning on radio last few months	v384a	Heard family planning on radio last few months	Categorical (0 “No”, 1 “Yes”	Categorical
Heard Family planning on TV last few months	v384b	Heard Family planning on TV last few months	Categorical (0 “No”, 1 “Yes”	Categorical
Heard family planning by text messages on mobile phone	v384d	Heard family planning by text messages on mobile phone	Categorical (0 “No”, 1 “Yes”	Categorical
Owns a mobile telephone	v169a	Owns a mobile telephone	Categorical (0 “No”, 1 “Yes”	Categorical
Use of internet	v171a	Use of internet	Categorical (0 “No”, 1 “Yes”	Categorical
Recent sexual activity	v536	Recent sexual activity	Categorical (“Active in last 4 weeks”, 2 “Not active in last 4 weeks—postpartum abstinence”, 3 “Not active in last 4 weeks—not postpartum abstinence”)	Categorical (recoded to v536_act: 1 “Active in last 4 weeks”, 2 “Not active in last 4 weeks”)
Sexual partners, in last 12 months	v766b	Number of sex partners, including spouse, in last 12 months	Continuous (0–95)	Categorical (recoded to sexual partners, in last 12 months: 0 “No partner”, 1 “1 partner”, 2 “2 or more partners”)
Modern contraceptive use	v364	Contraceptive use and intention	Categorical (1 “Using modern method”, 2 “Using traditional method”, 3 “Non-user—intends to use later”, 4 “Does not intend to use”)	Categorical (recoded to v364_cat “modern contraceptive use”: 0 “No” 1 “Yes”)

### Predictor variables

For the analysis, several potential predictors were selected based on the existing literature and their availability in the UDHS 2016 data set.

The study examines several predictor variables associated with modern contraceptive use among sexually active young women aged 15–24 years in Uganda. Age is categorized into (15–19) and (20–24). Region is divided into (Central, Eastern, Northern, and Western). Place of residence is classified as (Urban and Rural). Religion includes (Anglican, Catholic, Muslim, Pentecostal/Born Again, and Others). Highest level of education is categorized into (No education, Primary, Secondary, and Higher). Wealth index is classified into five levels: (Lowest, Second, Middle, Fourth, and Highest). Marital status is divided into (Never in union, Married, Living with a partner, and No longer living with a partner). Currently working is classified as (No and Yes). Parity refers to the number of children and is categorized into (No child, 1–2 children, and 3 or more children). Exposure to family planning messages is assessed through different media, including radio (No and Yes), television (No and Yes), and text messages on mobile phones (No and Yes). Mobile phone ownership is classified into (No and Yes). Use of the internet is categorized as (Never and Yes ever). Recent sexual activity is grouped into (Active last 4 weeks and Not active last 4 weeks). Sexual partners in the last 12 months include (No partner, 1 partner, and 2 or more partners). Knowledge of modern contraceptive methods is classified as (No and Yes).

Data was collected using female questionnaire and AGYW were asked if they were using any method of modern contraception. See Table 1 for the description of the outcome variable and all the predictor variables.

### Sample design

The 2016 UDHS used the sampling frame of the Uganda National Population and Housing Census (NPHC), conducted in 2014. The census frame is a complete list of all census enumeration areas (EAs) created for the 2014 NPHC. During the NPHC Uganda was divided administratively into 112 districts, these were grouped into 15 regions for this survey. Three special areas, the Lake Victoria islands, the mountain districts, and greater Kampala, were also considered.

The sample was stratified and selected in two stages. In the first stage, 697 EAs (162 and 535 EAs in urban and rural areas respectively) were selected from the 2014 Uganda NPHC. The second stage of sampling constituted households. In each of the 696 accessible selected EAs, a household listing was conducted, and to reduce the workload, any large EA with more than 300 households was segmented for the 2016 UDHS. A total of 20,880 households were selected for the 2016 UDHS, with 30 households randomly chosen from each enumeration area (EA) or EA segment to ensure representativeness.

### Sampling size calculation

A representative sample of 20,880 households was randomly selected for the 2016 Uganda DHS, with 19,088 eligible women being identified. Interviews were completed with 18,506 (97.0%) women and of 18,506 women, 8,058 were adolescent girls and young women aged 15–24. All adolescent girls who reported being currently pregnant (*n* = 926) were excluded from the analysis. Additionally, adolescent girls and young women who reported not being sexually active (*n* = 2,597) were also removed. One observation with missing data for the variable recent sexual activity was dropped. After these exclusions, the final sample of adolescent girls and young women who met the inclusion criteria for the analysis consisted of 4,534 participants.

### Inclusion and exclusion criteria

Adolescent girls and young women (AGYW) aged 15–24 years who were either permanent residents of the selected households or visitors who stayed in the household the night before the survey were eligible for inclusion in the analysis. AGYW who were pregnant, those who had never had sex, as well as those with missing data on key variables, were excluded from the study.

### Data analysis

#### Data management

The 2016 Uganda Demographic and Health Survey (UDHS) dataset was utilized for this study. Specifically, the women's dataset (UGIR7AFL.DTA) was selected, which includes data on women aged 15–49. The Standard Recode Manual for DHS7 was referenced to understand how the variables were collected and coded. To ensure data completeness, code booking and tabulation of the variables of interest were conducted based on the conceptual framework that was adapted from the Andersen (19) theoretical model of healthcare utilization. This process also helped in identifying the type and categories of variables, whether continuous or categorical, and their respective ranges or categories.

The outcome variable and all the potential predictor variables were checked for duplicates and any missing values before proceeding with data analysis. All variables with a lot of missing data were not considered in the analysis.

#### Sampling weights and probabilities

Due to the non-proportional allocation of the sample to different regions and their urban and rural areas, as well as the potential differences in response rates, sampling weights were used for any analysis using the 2016 UDHS data to ensure that the survey results were representative at both the national and domain levels.

#### Univariable analysis

The analysis began by declaring the survey design characteristics using the command “svyset” to account for complex survey design [weighting (wgt), clustering (v021), stratification (v023) were used]. Code booking, tabulation, renaming, and recoding of variables were conducted to prepare the data for analysis. Unweighted frequencies and percentages for the different categories of the variables were obtained through tabulation. Weighted percentages were then calculated by adding the “svy” prefix to the tabulation command, ensuring that the results were representative of the population.

#### Bivariable analysis

Weighted cross-tabulations of the outcome variable (modern contraceptive use) and each independent variable were performed to obtain weighted percentages of AGYW who used modern contraceptives and those who did not. The selection of the regression model for bivariable analysis was based on the prevalence of the outcome variable. Since the weighted prevalence of modern contraceptive use was 36.4%, which is relatively high, the logistic regression model was deemed inappropriate as it tends to overestimate measures of association for common outcomes. Instead, modified Poisson regression with robust variance was selected for bivariable analysis to obtain crude prevalence ratios (PRs), 95% confidence intervals (CIs), and corresponding p-values. The “svy” prefix was added to the modified Poisson regression command to obtain better estimates and the “vce” was linearized. A threshold p value of <0.25 was considered for selecting candidate predictor variables for multivariable model building.

#### Multivariable analysis

Multicollinearity among the independent variables was assessed using the command “corr.” Variables with a correlation coefficient of 0.4 or higher were considered highly correlated. For instance, wealth index was highly correlated with place of residence, education level and owning a mobile phone. Owning a mobile phone was also highly correlated with education level. Finally parity was highly correlated with marital status and age of respondent. Highly correlated variables were considered for inclusion in the model based on their stronger association with the outcome in the bivariable analysis.

The selection of the regression model for multivariable analysis was guided by the prevalence of the outcome variable. Modified Poisson regression with robust variance was performed for multivariable analysis using a “glm” function, and weights were incorporated to obtain accurate unbiased estimates of prevalence ratios, thus the “svy” prefix was not added to the modified Poisson regression command. Also, the model accounted for weighting through its robust variance estimation i.e., option “vce (robust)” was specified.

The stepwise logistic selection method was used to build the final multivariable model. This approach involved adding or removing variables step-by-step based on their statistical significance (*p* < 0.05) and contribution to the model. Variables with the least significance were removed iteratively until the most parsimonious model was achieved. The goodness of fit of the model was assessed using the Akaike Information Criterion (AIC). The final model, with an AIC of 1.307144, was selected as the best-fitting model. Adjusted prevalence ratios (aPRs), 95% confidence intervals (CIs), and corresponding p-values were obtained from this model. The significance level for independent variables was set at *p* < 0.05. All analyses were conducted using Stata Version 14.0.

## Results

### Background characteristics of sexually active adolescent girls and young women

Table 2 presents the socio-demographic characteristics of sexually active adolescent girls and young women aged 15–24 years in Uganda. The sample size comprised of 4,534 sexually active adolescent girls and young women (AGYW) and the majority of participants (64.42%) were aged 20–24, while 35.58% were aged 15–19. Geographically, the Central region had the highest representation (31.12%), followed by the Eastern (28.79%), Western (22.8%), and Northern regions (17.29%). Most participants resided in rural areas (72.98%), with only 27.02% living in urban areas.

**Table 2:** The socio-demographic characteristics of sexually active adolescent girls and young women (15–24).

∑*n* = 4,534			
Variable	Un weighted number	Un weighted percent	Weighted percent
Age			
15–19	1,617	35.66	35.58
20–24	2,917	64.34	64.42
Region			
Central	1,129	24.90	31.12
Eastern	1,440	31.76	28.79
Northern	895	19.74	17.29
Western	1,070	23.60	22.8
Place of residence			
Urban	1,105	24.37	27.02
Rural	3,429	75.63	72.98
Religion			
Anglican	1,376	30.79	30.75
Catholic	1,799	39.68	38.54
Muslim	633	13.96	15.32
Pentecostal/born again	578	12.75	12.79
Others	128	2.82	2.60
Highest level of education			
No education	172	3.79	2.89
Primary	2,676	59.02	56.20
Secondary	1,364	30.08	33.05
Higher	322	7.10	7.87
Wealth index combined			
Lowest	945	20.84	17.74
Second	937	20.67	19.34
Middle	770	16.98	16.5
Fourth	830	18.31	20.06
Highest	1,052	23.20	26.36
Marital status			
Never in union	1,538	33.92	34.14
Married	967	21.33	20.47
Living with a partner	1,578	34.80	34.95
No longer living with a partner	451	9.95	10.44
Currently working			
No	1,401	30.90	31.93
Yes	3,133	69.10	68.07
Parity			
No child	1,406	31.01	31.36
1–2 children	2,460	54.26	54.32
3 or more children	668	14.73	14.32
Heard family planning on radio last few months			
No	1,579	34.83	34.62
Yes	2,955	65.17	65.38
Heard Family planning on TV last few months			
No	3,630	80.06	78.25
Yes	904	19.94	21.75
Heard family planning by text messages on mobile phone			
No	4,387	96.76	96.68
Yes	147	3.24	3.32
Owns a mobile telephone			
No	2,705	59.66	56.71
Yes	1,829	40.34	43.29
Use of internet			
Never	3,960	87.34	86.26
Yes ever	574	12.66	13.74
Modern contraceptive use			
No	3,105	68.48	67.40
Yes	1,429	31.52	32.60
Recent sexual activity			
Active last 4 weeks	2,425	53.48	53.27
Not active last 4 weeks	2,109	46.52	46.73
Sexual partners, in last 12 months			
No partner	602	13.28	13.08
1 partner	3,752	82.75	82.91
2 or more partners	180	3.97	4.00
Knows modern method			
No	26	0.57	0.42
Yes	4,508	99.43	99.58

Religiously, Catholics constituted the largest group (38.54%), followed by Anglicans (30.75%), Muslims (15.32%), Pentecostal/Born Again (12.79%), and other religions (2.60%). Educationally, the majority had completed primary education (56.20%), while 33.05% had secondary education, 7.87% had higher education, and 2.89% had no formal education. Wealth distribution showed that 26.36% were in the highest wealth quintile, while 17.74% were in the lowest quintile.

Marital status revealed that 34.14% had never been in a union, 34.95% were living with a partner, 20.47% were married, and 10.44% were no longer living with a partner. Most participants (68.07%) were currently working, and 54.32% had 1–2 children, while 14.32% had three or more children. Media exposure was relatively high, with 65.38% having heard family planning messages on the radio and 21.75% on television. However, only 3.32% had received family planning messages via mobile text, and 43.29% owned a mobile phone. Internet usage was low, with only 13.74% having ever used the internet.

The modern contraceptive prevalence rate (mCPR) among AGYW was 32.60%, with 67.40% not using any modern contraceptive method. Recent sexual activity was reported by 53.27% of participants and 82.91% had one sexual partner in the last 12 months, while 4.00% had two or more partners. Nearly all participants (99.58%) were aware of modern contraceptive methods.

### Prevalence of modern contraceptive use by socio-demographic characteristics of sexually active adolescent girls and young women (15–24)

Table 3 shows the prevalence of modern contraceptive use according to various socio-demographic factors and their crude prevalence ratios (cPR). Age was a significant predictor, with AGYW aged 20–24 having a 1.5 times higher prevalence of modern contraceptive use compared to those aged 15–19 (PR: 1.503, 95% CI: 1.323–1.708). Regionally, AGYW in the Central region had the highest contraceptive use, while those in the Northern region had the lowest (PR: 0.632, 95% CI: 0.534–0.748). Urban residents were more likely to use modern contraceptives than rural residents were (PR: 0.812, 95% CI: 0.713–0.924).

**Table 3:** Prevalence of modern contraceptive use by socio-demographic characteristics of sexually active adolescent girls and young women (15–24) and the crude prevalence ratios (PRs).

Variable	Modern contraceptive use		Crude PR (95% CI)
	No (%)	Yes (%)	
Age			
15–19	1,216 (75.39)	397 (24.61)	1
20–24	1,840 (62.99)	1,081 (37.01)	1.503 (1.323–1.708)^a^
Region			
Central	852 (60.35)	559 (39.65)	1
Eastern	911 (69.78)	394 (30.22)	0.762 (0.665–0.873)^a^
Northern	588 (74.93)	197 (25.07)	0.632 (0.534–0.748)^a^
Western	706 (68.32)	327 (31.68)	0.799 (0.678–0.941)^b^
Place of residence			
Urban	762 (62.22)	463 (37.78)	1
Rural	2,294 (69.32)	1,015 (30.68)	0.812 (0.713–0.924)^b^
Religion			
Anglican	936 (67.15)	458 (32.85)	1
Catholic	1,199 (68.61)	549 (31.39)	0.955 (0.837–1.091)
Muslim	440 (63.36)	254 (36.64)	1.115 (0.943–1.319)
Pentecostal/born again	388 (66.86)	192 (33.14)	1.009 (0.854–1.191)
Others	93 (79.03)	25 (20.97)	0.638 (0.433–0.940)^c^
Highest level of education			
No education	109 (83.16)	22 (16.84)	1
Primary	1,792 (70.35)	755 (29.65)	1.761 (1.132–2.738)^b^
Secondary	912 (60.88)	586 (39.12)	2.323 (1.483–3.640)^a^
Higher	242 (67.97)	114 (32.03)	1.902 (1.197–3.023)^b^
Wealth index			
Lowest	624 (77.60)	180 (22.40)	1
Second	584 (66.64)	293 (33.36)	1.449 (1.251–1.770)^a^
Middle	505 (67.48)	243 (32.52)	1.452 (1.224–1.722)^a^
Fourth	592 (65.15)	317 (34.85)	1.556 (1.291–1.875)^a^
Highest	750 (62.77)	445 (37.23)	1.662 (1.381–1.999)^a^
Marital status			
Never in union	1,171 (75.64)	377 (24.36)	1
Married	589 (63.47)	339 (36.53)	1.499 (1.292–1.740)^a^
Living with a partner	989 (62.41)	596 (37.59)	1.543 (1.348–1.765)^a^
No longer living with a partner	307 (64.91)	166 (35.09)	1.440 (1.209–1.715)^a^
Currently working			
No	997 (68.86)	451 (31.14)	1
Yes	2,059 (66.72)	1,027 (33.28)	1.069 (0.960–1.190)
Parity			
No child	1,121 (78.84)	301 (21.16)	1
1–2 children	1,541 (62.56)	922 (37.44)	1.769 (1.527–2.049)^a^
3 or more children	394 (60.74)	255 (39.26)	1.855 (1.570–2.191)^a^
Heard family planning on radio			
No	1,118 (71.24)	451 (28.76)	1
Yes	1,938 (65.37)	1,026 (34.63)	1.204 (1.079–1,344)^b^
Heard Family planning on TV			
No	2,445 (68.93)	1,102 (31.07)	1
Yes	611 (61.92)	376 (38.08)	1.226 (1.074–1.398)^b^
Heard family planning by text messages on mobile phone			
No	2,975 (67.87)	1,409 (32.13)	1
Yes	81 (53.85)	69 (46.15)	1.436 (1.155–1.786)^b^
Owns a mobile telephone			
No	1,842 (71.62)	730 (28.38)	1
Yes	1,214 (61.87)	748 (38.13)	1.343 (1.209–1.492)^a^
Use of internet			
Never	2,643 (67.57)	1,268 (32.43)	1
Yes ever	413 (66.37)	210 (33.63)	1.037 (0.886–1.213)
Recent sexual activity			
Active last 4 weeks	1,398 (57.88)	1,017 (42.12)	1
Not active last 4 weeks	1,658 (78.25)	461 (21.75)	0.517 (0.464–0.574)^a^
Sexual partners, in last 12 months			
No partner	540 (91.08)	53 (8.92)	1
1 partner	2,409 (64.07)	1,351 (35.93)	4.030 (2.945–5.513)^a^
2 or more partners	107 (59.04)	74 (40.96)	4.594 (3.137–6.729)^a^

KEY: ^a^*P* < 0.001. ^b^*p* < 0.01. ^c^*p* < 0.05.

Education was strongly associated with contraceptive use, with AGYW who had primary, secondary and higher education being more likely to use modern contraceptives compared to those with no education (PR 1.761 95% CI:1.132–2.738; PR 2.323, 95% CI: 1.483–3.640; and PR: 1.902, 95% CI: 1.197–3.0230 respectively). Similarly, wealthier AGYW were more likely to use modern contraceptives, with those in the highest wealth quintile having a 1.7 times higher prevalence compared to the lowest quintile (PR: 1.662, 95% CI: 1.381–1.999).

Marital status also played a significant role, with married AGYW and those living with a partner having higher contraceptive use compared to those never in a union (PR: 1.499, 95% CI: 1.292–1.740 and PR: 1.543, 95% CI: 1.348–1.765, respectively). Parity was another significant factor, with AGYW who had 1–2 children or three or more children being more likely to use modern contraceptives compared to those with no children (PR: 1.769, 95% CI: 1.527–2.049 and PR: 1.855, 95% CI: 1.570–2.191, respectively).

Media exposure, particularly through radio and television, was associated with higher contraceptive use (PR: 1.204, 95% CI: 1.079–1.344 and PR: 1.226, 95% CI: 1.074–1.398, respectively). Mobile phone ownership also increased the likelihood of contraceptive use (PR: 1.343, 95% CI: 1.209–1.492). Recent sexual activity and having multiple sexual partners were also significant predictors of contraceptive use.

### Factors associated with modern contraceptive use among sexually active adolescent girls and young women (15–24)

Table 4 presents the key predictors of modern contraceptive use from the multivariable Poisson regression analysis. Regionally, AGYW in the Eastern, Northern, and Western regions had significantly lower contraceptive use compared to those in the Central region (aPR: 0.868, 95% CI: 0.78–0.994; aPR: 0.810, 95% CI: 0.689–0.952; and aPR: 0.837, 95% CI: 0.734–0.954, respectively). Education remained a strong predictor, with AGYW who had secondary or higher education being more than twice as likely to use modern contraceptives compared to those with no education (aPR: 2.304, 95% CI: 1.472–3.605 and aPR: 2.220, 95% CI: 1.373–3.558, respectively).

**Table 4:** Weighted modified poisson regression estimates for modern contraceptive use by socio-demographic characteristics of sexually active adolescent girls and young women (15–24).

Variable	Modern contraceptive use		Crude PR (95% CI)	Adjusted PR (95% CI)
	No (%)	Yes (%)		
Region				
Central	852 (60.35)	559 (39.65)	1	1
Eastern	911 (69.78)	394 (30.22)	0.762 (0.665–0.873)^a^	0.868 (0.78–0.994)^b^
Northern	588 (74.93)	197 (25.07)	0.632 (0.534–0.748)^a^	0.810 (0.689–0.952)^b^
Western	706 (68.32)	327 (31.68)	0.799 (0.678–0.941)^b^	0.837 (0.734–0.954)^b^
Highest level of education				
No education	109 (83.16)	22 (16.84)	1	1
Primary	1,792 (70.35)	755 (29.65)	1.761 (1.132–2.738)^b^	1.762 (1.135–2.736)^b^
Secondary	912 (60.88)	586 (39.12)	2.323 (1.483–3.640)^a^	2.304 (1.472–3.605)^a^
Higher	242 (67.97)	114 (32.03)	1.902 (1.197–3.0,230^b^	2.220 (1.373–3.558)^b^
Wealth index				
Lowest	624 (77.60)	180 (22.40)	1	1
Second	584 (66.64)	293 (33.36)	1.449 (1.251–1.770)^a^	1.405 (1.188–1.662)^a^
Middle	505 (67.48)	243 (32.52)	1.452 (1.224–1.722)^a^	1.389 (1.556–1.669)^a^
Fourth	592 (65.15)	317 (34.85)	1.556 (1.291–1.875)^a^	1.423 (1.185–1.710)^a^
Highest	750 (62.77)	445 (37.23)	1.662 (1.381–1.999)^a^	1.463 (1.204–1.777)^a^
Parity				
No child	1,121 (78.84)	301 (21.16)	1	1
1–2 children	1,541 (62.56)	922 (37.44)	1.769 (1.527–2.049)^a^	1.627 (1.422–1.860)^a^
3 or more children	394 (60.74)	255 (39.26)	1.855 (1.570–2.191)^a^	1.744 (1.476–2.061)^a^
Heard family planning by text messages on mobile phone				
No	2,975 (67.87)	1,409 (32.13)	1	1
Yes	81 (53.85)	69 (46.15)	1.436 (1.155–1.786)^b^	1.325 (1.071–1.639)^a^
Recent sexual activity				
Active last 4 weeks	1,398 (57.88)	1,017 (42.12)	1	1
Not active last 4 weeks	1,658 (78.25)	461 (21.75)	0.517 (0.464–0.574)^a^	0.663 (0.592–0.742)^a^
Sexual partners, in last 12 months				
No partner	540 (91.08)	53 (8.92)	1	1
1 partner	2,409 (64.07)	1,351 (35.93)	4.030 (2.945–5.513)^a^	2.828 (2.054–3.895)^a^
2 or more partners	107 (59.04)	74 (40.96)	4.594 (3.137–6.729)^a^	3.205 (2.212–4.644)^a^

KEY: ^a^*P* < 0.001. ^b^*p* < 0.01. ^c^*p* < 0.05.

Goodness of fit test.

AIC = 1.307144.
scalar dev = e(deviance) scalar df = e(df) display “GOF casewise” “G^2^ = “dev” df = “df” *p*-value =” chiprob(df,dev)

GOF casewise G^2^ = 2,936.347 df = 4,517 p-value = 1.

Wealth also remained significant, with AGYW in the highest wealth quintile having a 1.5 times higher prevalence of contraceptive use compared to those in the lowest quintile (aPR: 1.463, 95% CI: 1.204–1.777). Parity was another key predictor, with AGYW who had 1–2 children or three or more children being more likely to use modern contraceptives compared to those with no children (aPR: 1.627, 95% CI: 1.422–1.860 and aPR: 1.744, 95% CI: 1.476–2.061, respectively).

Media exposure through mobile text messages was associated with higher contraceptive use (aPR: 1.325, 95% CI: 1.071–1.639). Recent sexual activity and having multiple sexual partners were also significant predictors, with AGYW who were sexually active in the last four weeks and those with two or more partners being more likely to use modern contraceptives (aPR: 0.663, 95% CI: 0.592–0.742 and aPR: 3.205, 95% CI: 2.212–4.644, respectively).

## Discussion of results

This study aimed to determine the factors associated with modern contraceptive use among sexually active adolescent girls and young women (AGYW) aged 15–24 in Uganda, using data from the 2016 Uganda Demographic and Health Survey (UDHS). The analysis revealed that 32.6% of AGYW were using modern contraceptive methods. The age specific modern contraceptive rate (mCPR) was 8.8% for adolescent girls (15–19) and 23.8% for young women (20–24). These finding highlight a significant gaps in contraceptive uptake among this vulnerable population.

The general rate of 32.6% is slightly higher than the 27% reported in a similar study across sub-Saharan Africa (1), while the age specific mCPR of 8.8% is lower than the 9.4% reported in a similar study (15). The low utilization rate may be attributed to several factors, including limited access to youth-friendly family planning services, cultural and religious norms that discourage contraceptive use, and misinformation or fear of side effects associated with modern methods (1, 15).

The study found that education and wealth index were significant predictors of modern contraceptive use. Education was a strong predictor of contraceptive use, with AGYW who had primary, secondary or higher education being more likely to use modern contraceptives compared to those with no education. This finding is consistent with studies in Ethiopia, Uganda and, South Africa which have shown that level of education empowers women to make informed reproductive health decisions (2, 13, 14).

Wealthier AGYW were also more likely to use modern contraceptives, reflecting better access to healthcare services and the ability to afford contraceptive methods. This is similar to previous studies from Kenya, South Africa and Burkina Faso that found that women from wealthier backgrounds were more likely to use contraception (2, 20, 21).

Regional disparities in contraceptive use were evident, with AGYW in the Central region reporting the highest utilization, followed by those in the Eastern and Western regions, while the Northern region had the lowest. AGYW from regions outside Central were significantly less likely to use modern contraceptives. These differences may reflect broader socio-cultural, economic, and health system factors that vary across Uganda's regions. For example, the Northern region has historically faced prolonged conflict, which has disrupted health service delivery and limited access to reproductive health information and services (22). Additionally, traditional norms in some Northern communities may discourage contraceptive use among unmarried and young women, emphasizing fertility and early childbearing as social expectations (15). Economic disparities also play a role; AGYW in Northern Uganda often have fewer employment and educational opportunities, which can reduce autonomy in reproductive health decision-making compared to their peers in the Central region (15). Furthermore, the Central region benefits from higher urbanization and better infrastructure, increasing access to family planning facilities and commodities. Several other studies have found similar region disparities in contraceptive use and these observed regional differences in utilization of modern contraceptives could be attributed to the differences in access to modern contraceptives, sociocultural contexts and job opportunities available to AGYW in the different regions (1, 14, 15).

Media exposure, particularly through mobile text messages, was associated with higher contraceptive use, with those exposed more likely to use contraceptives compared to those who were not. This highlights the role of mass media in spreading information and increasing awareness and positive attitudes toward contraception use as seen in studies from Nigeria and Kenya (18, 20). However, this result should be interpreted cautiously given the very low level of exposure observed in our sample (3.32%), which suggests limited population reach. Structural and equity-related barriers, such as disparities in mobile phone ownership, shared device use, network access, and privacy concerns, may restrict the scalability and inclusiveness of SMS-based interventions, particularly among younger, rural, and socioeconomically disadvantaged AGYW. Therefore, while digital messaging platforms show promise as channels for reproductive health communication, they should be considered complementary to, rather than substitutes for, broader community-based and health system–integrated strategies designed to ensure equitable access to family planning information and services.

Parity was a significant predictor of contraceptive use, with AGYW who had 1–2 children or three or more children being more likely to use modern contraceptives compared to those with no children. This is consistent with studies in Uganda and Ghana, which suggest that women with more children are more likely to use contraceptives to limit future pregnancies (14, 23).

Recent sexual activity and having multiple sexual partners were also significant predictors of contraceptive use, reflecting the need for family planning services among sexually active AGYW (24–26).

### Strengths

The study utilized data from the Uganda Demographic and Health Survey (UDHS) 2016, which is a nationally representative survey. This ensures that the findings are generalizable to the entire population of sexually active adolescent girls and young women (AGYW) aged 15–24 in Uganda.

The study employed a robust analytical approach, including univariable, bivariable, and multivariable modified Poisson regression analyses while adjusting for survey design characteristics. This allowed for obtaining accurate estimates.

The study examined a wide range of socio-demographic, cultural, and structural factors, providing a holistic understanding of the determinants of modern contraceptive use among AGYW in Uganda.

### Study limitations

The study relied on cross-sectional data, which limits the ability to establish causal relationships between the independent variables and modern contraceptive use. Longitudinal studies are needed to assess trends and the long-term impact of interventions.

The data on modern contraceptive use and other variables were self-reported, which may be subject to recall bias and social desirability bias.

Limited analysis of the male involvement in contraceptive use. Though the study highlighted the importance of marital status and living with a partner, it did not specifically explore the role of male partners in shaping contraceptive decisions. Male involvement is a critical factor in family planning and should be addressed in future research.

Although the study identified regional disparities in contraceptive use, it did not explore the underlying reasons for these disparities in detail. Future research should investigate the specific cultural, economic, and healthcare access issues that contribute to regional differences.

Finally, although this study used the most recent publicly accessible UDHS dataset suitable for analysis, the data were collected in 2016. More recent datasets (e.g., UDHS 2022) were not yet available for public secondary analysis at the time of this study. As a result, the findings may not fully reflect recent changes in contraceptive programs, policies, or sociocultural dynamics in Uganda. Future analyses using updated datasets are therefore recommended to validate and extend these findings.

## Conclusion

This study provides a comprehensive analysis of the factors associated with modern contraceptive use among sexually active adolescent girls and young women (AGYW) aged 15–24 in Uganda. The findings reveal that the modern contraceptive prevalence rate (mCPR) among AGYW is 32.60%, indicating that a significant proportion of this population remains at risk of unintended pregnancies, maternal mortality, and other adverse reproductive health outcomes. Key predictors of modern contraceptive use include education, wealth, parity, media exposure, sexual partners and recent sexual activity. Regional disparities were also evident, with AGYW in the Central region having the highest contraceptive use, while those in the Northern region had the lowest.

The study highlights the critical role of education, wealth, and media exposure in empowering AGYW to make informed reproductive health decisions. It also underscores the importance of addressing cultural and structural barriers, such as limited access to healthcare services to improve contraceptive uptake among AGYW. Given similarities in demographic structure, health system organization, and sociocultural norms across several East African countries, these findings are likely most transferable to settings with comparable adolescent fertility patterns, levels of contraceptive access, and socioeconomic inequalities. In particular, countries with similar rural–urban disparities, regional development gaps, and youth reproductive health challenges may observe comparable associations. However, transferability may be limited in contexts that differ substantially from Uganda.

While the study has several strengths, including the use of nationally representative data and a robust analytical approach, it also has limitations, the cross-sectional design precludes causal inference, and reliance on self-reported data may introduce reporting bias. In addition, the analysis could not fully capture contextual or relational influences, such as partner dynamics or community-level norms, which may further affect contraceptive use.

## Recommendations

Based on the study findings the recommendations below have been proposed to improve modern contraceptive use among young adolescents and young women in Uganda:

Given the lower contraceptive use among AGYW aged 15–19, there is a need for targeted interventions to address the unique reproductive health needs of this group. This could include school-based sexual and reproductive health (SRH) education programs and youth-friendly family planning services.

Regional disparities in contraceptive use highlight the need for tailored interventions to address the specific cultural, economic, and healthcare access issues in different regions. For example, in the Northern region, where contraceptive use is lowest, interventions should focus on improving access to healthcare services and addressing cultural norms that discourage contraceptive use.

Media exposure, particularly through mobile text messages, was associated with higher contraceptive use, suggesting that digital communication platforms may be useful channels for promoting awareness and positive attitudes toward modern contraceptives. However, given the low exposure observed and potential inequities in access to mobile technology, such approaches should be implemented cautiously and as part of a broader, integrated strategies.

Lastly, future research should build on these findings by incorporating longitudinal designs to assess trends, the long-term impact of interventions and male involvement in contraceptive use. Qualitative research is also needed to explore the lived experiences of AGYW and the contextual factors influencing their reproductive health decisions.

## Data Availability

Publicly available datasets were analyzed in this study. This data can be found here: Demographic and Health Surveys repository, https://dhsprogram.com/Data/.
